# Extraction, Preconcentration and Isolation of Flavonoids from *Apocynum* *venetum* L. Leaves Using Ionic Liquid-Based Ultrasonic-Assisted Extraction Coupled with an Aqueous Biphasic System

**DOI:** 10.3390/molecules21030262

**Published:** 2016-03-04

**Authors:** Zhijian Tan, Yongjian Yi, Hongying Wang, Wanlai Zhou, Chaoyun Wang

**Affiliations:** Institute of Bast Fiber Crops, Chinese Academy of Agricultural Sciences, Changsha 410205, China; ibfcyyj@163.com (Y.Y.); Cswhy328@126.com (H.W.); aruofly@126.com (W.Z.)

**Keywords:** ionic liquids, ultrasonic-assisted extraction, flavonoids, aqueous biphasic system, isolation

## Abstract

*Background*: Ionic liquids (ILs) are considered as green solvents, and widely applied for the extraction of various compounds. *Methods*: The present research focuses on the extraction of flavonoids from *Apocynum* *venetum* L. leaves by ultrasound-assisted extraction (UAE). Several major influencing factors were optimized. Then, an aqueous biphasic system (ABS) was applied for further isolation of flavonoids. *Results*: The flavonoids were mainly distributed in the top phase, while impurities were extracted to the bottom phase. The parameters influencing the extraction, namely type and concentration of salt, temperature, and pH, were studied in detail. Under optimized conditions (72.43% IL extract, 28.57% (NH_4_)_2_SO_4_, 25 °C temperature, pH 4.5), the preconcentration factor and extraction efficiency were found to be 3.78% and 93.35%, respectively. *Conclusions*: This simple and efficient methodology is expected to see great use in the extraction and isolation of pharmaceutically active components from medicinal plant resources.

## 1. Introduction

*Apocynum* *venetum* L., belonging to the family Apocynaceae, is a traditional medicinal plant distributed throughout mid- and north-western China, mainly in the Xinjiang Uygur Autonomous Region. *A. venetum* has several pharmacological effects such as lowering blood pressure, antidepressant, anti-anxiety, antinephritis and antineurasthenia [1,2,3,4]. Tea prepared from *A. venetum* leaves has gained popularity as a healthy beverage that acts as an anti-aging nutritional supplement [5]. The flavonoids present as the major active constitutes in *A. venetum* leaf are responsible for the pharmacological effect of lowering blood pressure, scavenging free radicals and anti-inflammatory action [6,7,8].

Room temperature ionic liquids (ILs) are molten salts with a melting point below 100 °C. Usually, they are composed of organic cations and inorganic anions. As opposed to water and conventional volatile organic solvents, ILs have unique features of high ionic conductivity, wide electrochemical windows, negligible volatility, and high polarity and chemical stability [9,10,11,12]. In addition, some other assisted technologies coupled with ILs, such as ionic liquid-based ultrasonic-assisted extraction (IL-UAE) [13], ionic liquid-based microwave-assisted extraction (IL-MAE) [14], and ionic liquid-based negative pressure cavitation-assisted extraction (IL-NPCE) [15], provide remarkable advantages in the extraction and separation field. In particular, IL-UAE is an innovative technology combining IL and ultrasonic extraction, which can produce clear synergies in some cases [16]. In the field of extraction of medicinal components from different herb sources, IL-UAE has been reported to be a comparatively more effective method for the extraction of various compounds, such as aesculin and aesculetin from *Cortex fraxini* [17], piperine from white pepper [18], ginsenosides from ginseng root [19], alkaloids from *Catharanthus* *roseus* [20], biphenyl cyclooctene lignans from *Schisandra* *chinensis* Baill [21], magnolol and honokiol from *Magnoliae officinalis* cortex [22]. Aqueous biphasic systems based on ionic liquids (IL-ABS) were proposed in 2003 by Rogers [23], and have been developed to extract and isolate several bioactive compounds, such as anthraquinones [24], gallic acid [25], puerarin [26], and alkaloids [27].`

**Figure 1:**
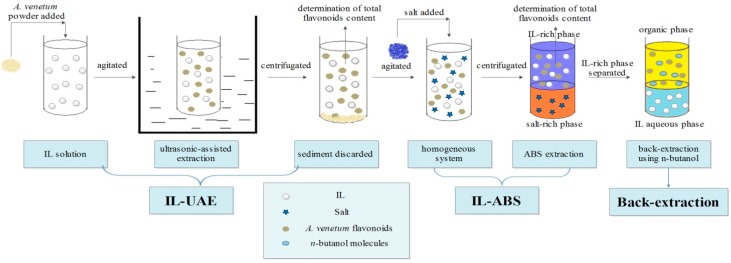
Process flowchart for the extraction and purification of flavonoids from *Apocynum* *venetum* L. using IL-UAE coupled with IL-ABS and back-extraction.

The analysis and identification of the flavonoids in *A. venetum* leaves using liquid chromatography-tandem mass spectrometry (LC-MS/MS) are well reported in the literature [28,29,30]. The present article reports the extraction of flavonoids from *A. venetum* into IL aqueous solution by the IL-UAE method, with further application of IL-ABS for preconcentration and purification of the flavonoids. Some factors affecting UAE were optimized by response surface methodology (RSM) to obtain the optimal extraction yields. The influencing parameters in IL-ABS were also investigated to obtain the maximal extraction efficiencies. The process flowchart for extraction and purification of flavonoids from *A. venetum* L. using IL-UAE coupled with IL-ABS and back-extraction is shown in Figure 1.

## 2. Results and Discussion

### 2.1. Selection of Ionic Liquid

**Figure 2:**
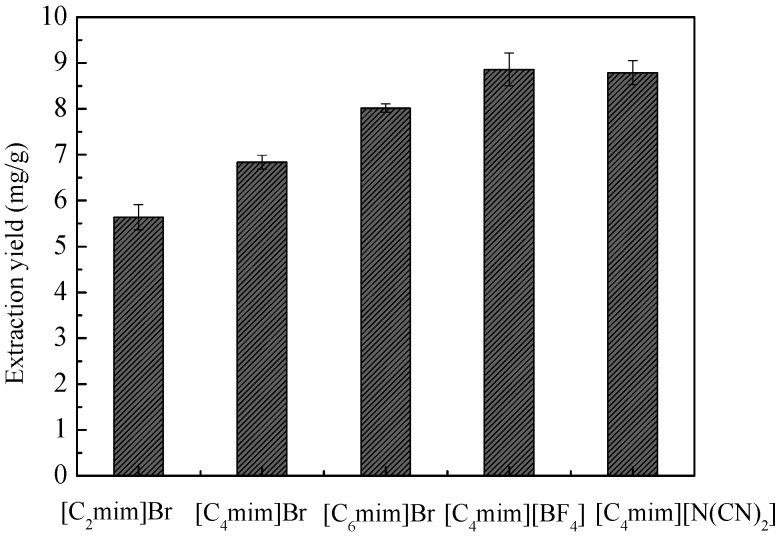
UAE of total flavonoids from *A. venetum* L. leaves using different ILs.

Commonly used ILs such as [C_n_mim]Br (*n* = 2, 4, and 6), [C_4_mim][BF_4_], and [C_4_mim][N(CN)_2_] were considered as the extractants for the present study. From Figure 2, it can be observed that both [C_4_mim][BF_4_] and [C_4_mim][N(CN)_2_] exhibited higher extraction yields than other ILs used in this IL-UAE study. The probable reason for this is that the extraction ability is related to the hydrophobicity of ILs, usually increasing with their increasing hydrophobicity. The order of hydrophobicity for the five studied ILs is as follows: [C_2_mim]Br < [C_4_mim]Br < [C_6_mim]Br < [C_4_mim][N(CN)_2_] < [C_4_mim][BF_4_]. In addition, it was reported that the factors influencing the extraction ability are very complicated, hydrogen-bonding ability can be the main factor influencing the extraction considering the anions [31], π-π and *n*-π interactions also should not be omitted in view of imidazole ring- containing cations [32]. Although [C_4_mim]BF_4_ was commonly used as the extractant in IL-UAE [18,33,34,35], some research groups had reported that tetrafluoroborate-based ILs were not water-stable as they could undergo hydrolysis in aqueous solution [36,37]. Thus, [C_4_mim][N(CN)_2_] was selected for further study. In general, [C_4_mim][N(CN)_2_] has comparatively lower viscosity than the other ILs used in the present study, about 29.3 mPa·s at 298.15 K and 0.101 MPa [38].

### 2.2. Optimization of UAE

#### 2.2.1. Single Factor Analysis

**Figure 3:**
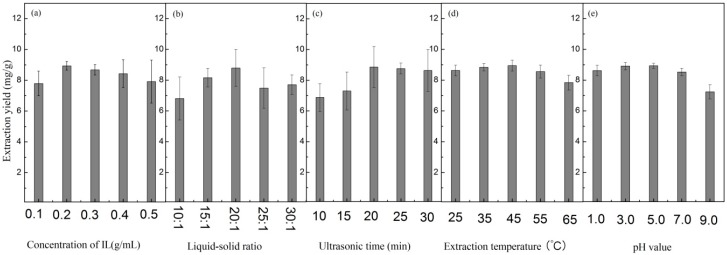
The effect of (**a**) IL concentration (the extraction conditions were 20:1 liquid/solid ratio, 30 min ultrasonic time at room temperature with pH being not adjusted); (**b**) liquid/solid ratio (the extraction conditions were 0.2 g/mL IL concentration, 30 min ultrasonic time at room temperature with pH being not adjusted); (**c**) ultrasonic time (the extraction conditions were 0.2 g/mL IL concentration, 20:1 liquid/solid ratio at room temperature with pH being not adjusted); (**d**) extraction temperature (the extraction conditions were 0.2 g/mL IL concentration, 20:1 liquid/solid ratio, 30 min ultrasonic time with pH being not adjusted); and (**e**) pH value (the extraction conditions were 0.2 g/mL IL concentration, 30 min ultrasonic time, 20:1 liquid/solid ratio at room temperature) on the extraction yields by UAE.

The factors such as IL concentration, liquid/solid ratio, ultrasonic time, extraction temperature, and pH can influence the extraction, hence, we had to optimize these conditions in order to obtain the maximal flavonoids extraction yield. The effect of aqueous solutions with different [C_4_mim][N(CN)_2_] concentrations (from 0.1 to 0.5 g/mL) was studied to find the optimal concentration for IL-UAE. As shown in Figure 3a, the maximal extraction yield was obtained at 0.2 g/mL IL concentration. Thus, the concentration of 0.2 g/mL was selected considering to obtain the maximum extraction yield and reduce operational cost.

Liquid/solid ratio (volume of extraction solvent/amount of dried *A. venetum* L. leaves) is another factor affecting the extraction. Employing a higher liquid/solid ratio may result in excess consumption of ILs, while a lower ratio may lead to incomplete extraction of flavonoids; hence, it is important to optimize the liquid/solid ratio. For this purpose, the influence of liquid/solid ratios of 10:1–30:1 mL/g on the extraction yield was investigated. The results in Figure 3b revealed that the maximal extraction yield was obtained at the liquid/solid ratio of 20:1. Hence, this value was selected for further study.

Ultrasound exposure time can also affect the UAE to some extent; hence, its optimization is essential to ensure complete extraction. The effect of ultrasonic irradiation times ranging from 10 to 30 min was studied. From Figure 3c, it can be observed that 20 min was sufficient for the extraction of flavonoids.

The effect of extraction temperature in the range of 25–65 °C was investigated. As shown in Figure 3d, the extraction yields varied little at temperatures below 55 °C, thus, the extraction was performed at room temperature.

Na_2_HPO_4_-H_3_PO_4_ buffer solution coupled with hydrochloric acid and sodium hydroxide solutions were applied to adjust the pH. The effect of pH in the range of 1.0–9.0 was studied. The results in Figure 3e indicated that the extraction yields varied little at pHs below pH 7.0. Thus, the extraction was performed under acidic conditions.

#### 2.2.2. Response Surface Methodology

To study the mutual influence among these factors, a Design-Expert (DE) 7.0 software (Statease Inc., Minneapolis, MN, USA) was applied to optimize the experimental conditions. Three factors with three levels—IL concentration (0.2, 0.3 and 0.4 g/mL), liquid/solid ratio (15:1, 20:1 and 25:1), and ultrasonic time (15, 20 and 25 min)—were developed on the basis of Box-Benhnken experimental design. The experimental conditions of different runs and the results for extraction yield were shown in Table 1. The experimental data were analyzed by multiple regressions to fit the second order regression equation. The regression model in the form of marked factors as an equation of independent variables was obtained: *Y* = 8.81− 0.73A + 0.21B − 0.24C − 0.16AB + 0.26AC + 0.067BC − 1.10A^2^ − 0.33B^2^ − 0.73C^2^ (*R*^2^ = 0.9947), *Y* represented the extraction yield, the marked factors A, B, and C represented IL concentration, liquid/solid ratio, and ultrasonic time, respectively.

Table 2 shows that the coefficient (*R*^2^) obtained from the calculated model was 0.9947, which implied that over 99.47% of the variations could be explained by this model, and the value of “Lack of fit” was not significant (*p* > 0.05). The Model *F*-value was 147.00 and the *p*-value was <0.0001 implied the fact that this model was significant, there was 0.01% probablity for “Model *F*-value” occuring owing to noise. All of the results above demonstrated that the model was acceptable. The value of “Prob > *F*” was less than 0.05, indicating the significance of the model terms. On this occasion, the effects of linear terms A, B, and C, all quadratic terms, and two interaction terms (AB and AC) were significant model.

**Figure 4:**
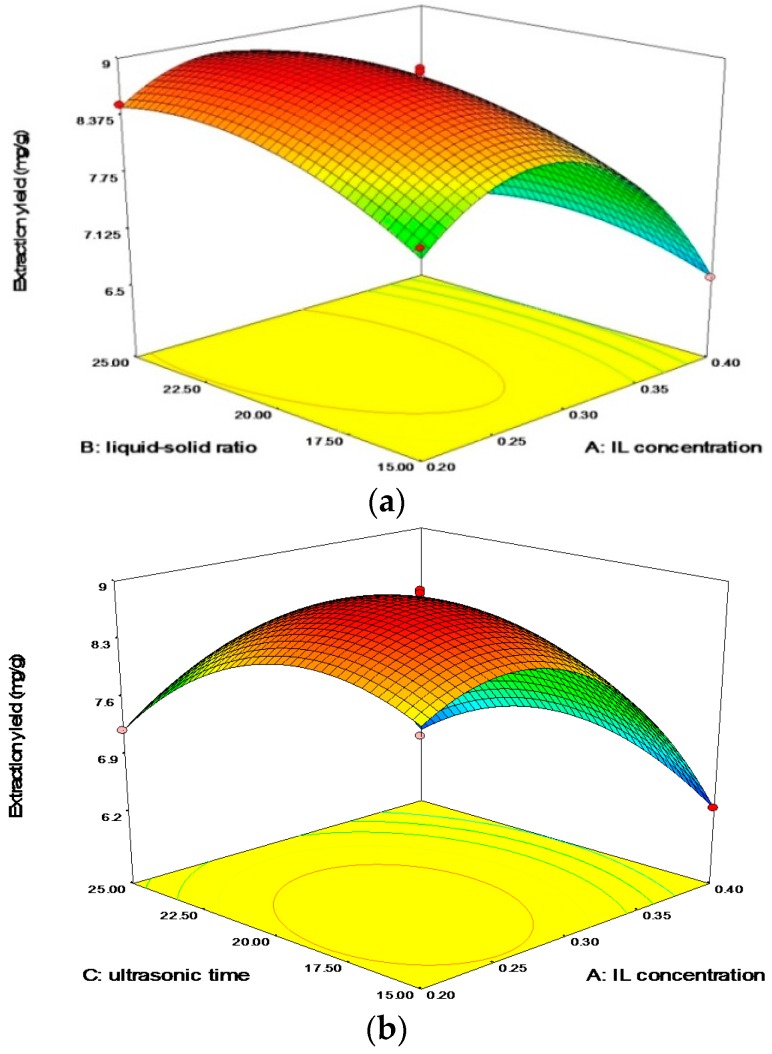

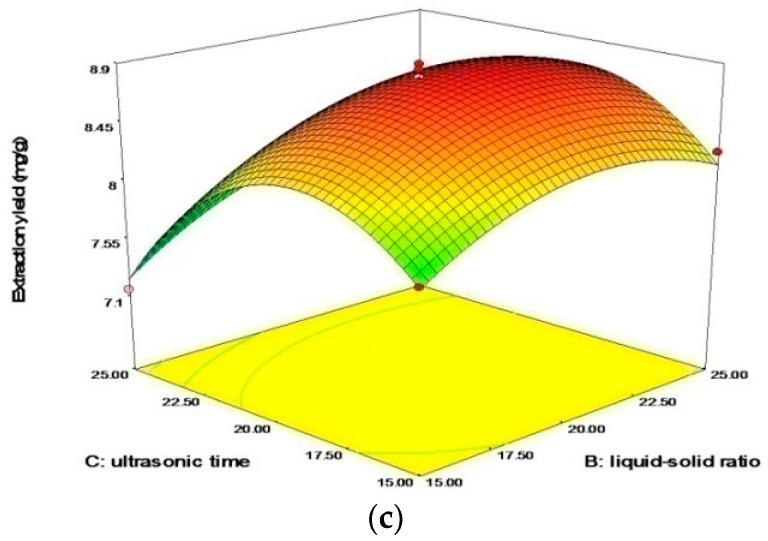
The plots for 3D response surface revealed the effect of (**a**) IL concentration and liquid/solid ratio; (**b**) IL concentration and ultrasonic time; (**c**) liquid/solid ratio and ultrasonic time on the extraction yields of total flavonoids.

Figure 4 showed the 3D response surfaces for the mutual interaction between IL concentration, liquid/solid ratio, and ultrasonic time. The optimum conditions proposed by DE software were as follows: IL concentration was 0.26 g/mL; liquid/solid ratio was 21.98:1; and ultrasonic time was 18.93 min. Under the practical conditions for use these values (0.26 g/mL IL concentration, 22:1 liquid/solid ratio; and 19 min ultrasonic irradiation time) in routine applications, the experiments were repeated 3 times. The realistic experimental value (8.92 mg/g) was satisfactorily close to the predicted value (9.02 mg/g). The results of verification experiments proved that this model can well reflect the expected optimization.

### 2.3. Comparison of Different Extraction Methods

IL-UAE was compared to other methods used for the extraction of flavonoids, such as heat reflux extraction using ILs [C_4_mim][N(CN)_2_] (IL-HRE), Soxhlet extraction using ethanol (Ethanol-SE), and UAE using ethanol (Ethanol-UAE). The liquid/solid ratios for all extraction methods are 30:1. It can be seen in Table 3 that the IL-UAE method gave the highest extraction yield in a shorter time and under milder temperature conditions. Therefore, the replacement of traditional volatile organic solvents by ionic liquid is feasible, moreover, the UAE is an effective auxiliary method in solvent extraction.

### 2.4. Extraction and Purification of Flavonoids by IL-ABS

**Figure 5:**
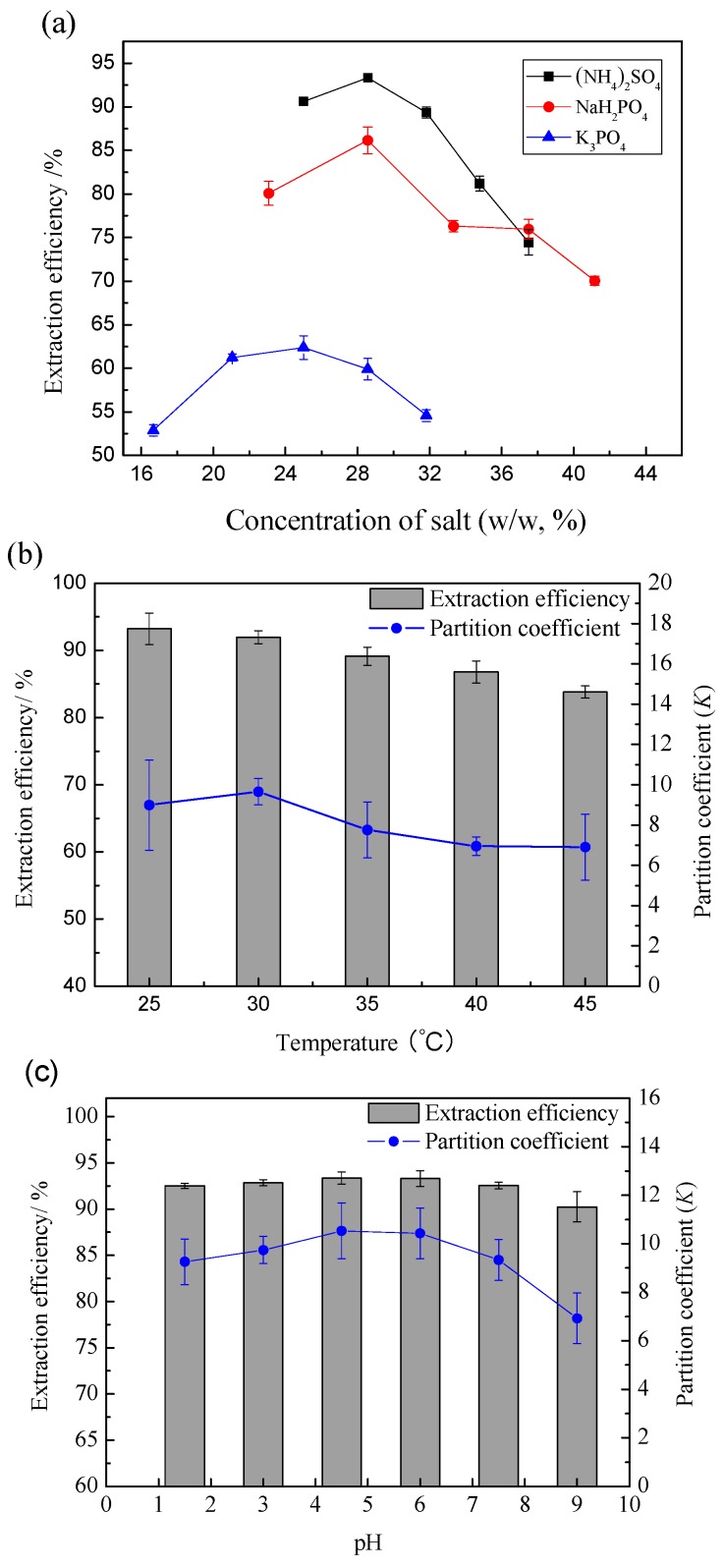
Effect of (**a**) salt type and concentration (each IL-ABS contains a given mass of IL extract and salt at room temperature); (**b**) extraction temperature; (**c**) system pH on the extraction efficiencies.

For further extraction and purification of flavonoids, ABS was formed by adding different salts to the IL extract. To obtain the optimum extraction conditions of IL-ABS extraction, the major factors influencing the extraction, such as salt type and concentration, extraction temperature, and system pH were studied. Three types of salts—NaH_2_PO_4_, (NH_4_)_2_SO_4_, and K_3_PO_4_—were chosen as the components to form ABS. The extraction efficiencies of ABSs formed by these salts are shown in Figure 5a. It can be seen that the system formed by (NH_4_)_2_SO_4_ had the best extraction ability among the three cases. The optimum extraction efficiency was obtained when the system was formed with 3.0 g IL extract (72.43%, *w*/*w*) and 1.2 g (NH_4_)_2_SO_4_ (28.57%, *w*/*w*). As the obtained flavonoids were acidic, 28.57% (NH_4_)_2_SO_4_was chosen as the optimal salt concentration to ensure stability under acidic conditions.

The partitioning of flavonoids in [C_4_mim][N(CN)_2_]/(NH_4_)_2_SO_4_ ABS was investigated in the 25–45 °C temperature range and at a constant pH value. From Figure 5b, it can be seen that the extraction efficiencies decreased as the temperature increased. The maximum extraction efficiency of 93.22% was obtained at 25 °C. Thus, the operation can be conducted at room temperature without heating, which benefits both the cost reduction of the process and the stability of the flavonoids.

To study the effect of pH, pH values of 1.5–9.0 were developed in [C_4_mim][N(CN)_2_]/(NH_4_)_2_SO_4_ ABS. As can be seen from Figure 5c, a notable extraction efficiency was obtained under acidic conditions with a maximum of 93.35% at pH 4.5, but no obvious difference was observed at pH 1.5–6.0. As the IL/(NH_4_)_2_SO_4_ABS has an optimum pH of 2.95, the system pH needed not be adjusted. Under the optimized conditions, a preconcentration factor of 3.78 for total flavonoids was obtained.

### 2.5. Isolation of Flavonoids and Recovery of IL

**Figure 6:**
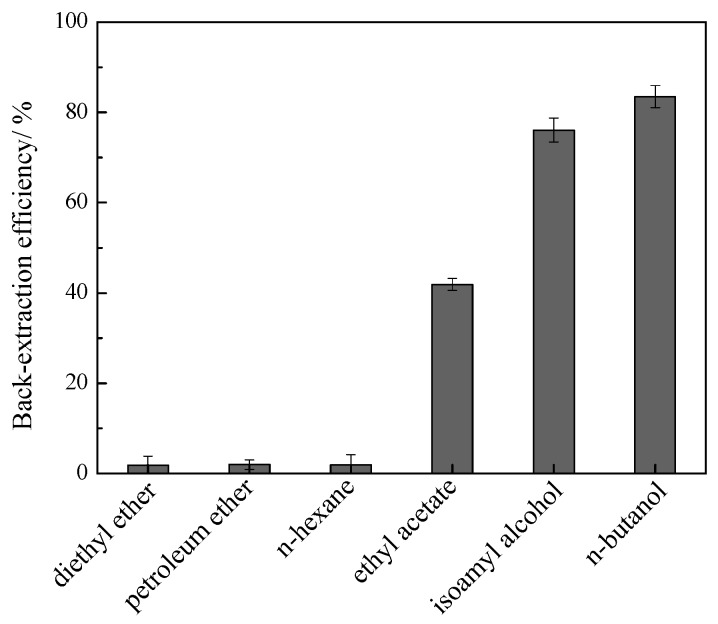
The back-extraction efficiencies for flavonoids using different organic solvents.

The results (Figure 6) for back-extraction showed that the maximum back-extraction efficiency (83.5% for total flavonoids) was obtained using *n*-butanol; dichloromethane and chloroform had no back-extraction ability, diethyl ether, petroleum ether and *n*-hexane had much lower back-extraction efficiencies. The organic alcohols thus had higher back-extraction efficiencies than other solvents; this is because alcohols have stronger hydrogen bonding interactions with flavonoids and water molecules, resulting in the flavonoids and water migrating to the organic layer from the IL layer. The IL can be reused after removal of water. The recovered IL was used for new extraction, and satisfactory extraction efficiency results were obtained (91.35%), the purity of recovered IL is 92.62% after further treatments.

## 3. Materials and Methods 

### 3.1. Materials and Reagents

*A. venetum* L. leaves were purchased from Hunan Xin Shen Zhi Lin Chinese Herbal Medicine Co. Ltd., (Changsha, China). The leaves were dried at 50 °C then crushed to powder of 60 mesh (250 μm) particle size. Five imidazolium ILs used in this study of [C_n_mim]Br (*n* = 2, 4, and 6, 1-alkyl-3-methylimidazolium bromide), 1-butyl-3-methylimidazoliumte trafluoroborate ([C_4_mim][BF_4_]), and 1-butyl-3-methylimidazoliumte dicyanamide ([C_4_mim][N(CN)_2_]) were synthesized using reported procedures [39,40]. The rutin standard (HPLC purity > 98%) was acquired from Aladdin Industrial Corporation (Shanghai, China). The phase-forming salts NaH_2_PO_4_·2H_2_O and (NH_4_)_2_SO_4_ (analytical grade) were purchased from Sinopharm Chemical Reagent Co., Ltd. (Shanghai, China). All other chemicals were of reagent-grade quality and used without further treatment.

### 3.2. Ultrasonic-Assisted Extraction

The IL aqueous solution was used for UAE of flavonoids. To a tube, a certain amount of *A. venetum* leaf powder and IL aqueous solution were added to a certain liquid/solid ratio, and the tube was placed in a KQ-5200DE ultrasonic instrument (Kunshan Ultrasound Co. Ltd., Jiangsu, China) at 30 °C for extraction of flavonoids in a certain time. The electric power and generators frequency is 200 W and 40 kHz, respectively. Then, the tubes were centrifuged at 10,000× *g* for 10 min to remove the sediment. The supernatant was separated for further analysis of the total flavonoids content. The NaNO_2_-Al(NO_3_)_3_-NaOH colorimetry method reported by Ozsoy [41] was used to determine the total flavonoids content at 510 nm using UV-2100 spectrophotometer (Unico, Dayton, NJ, USA). The calibration curve for the flavonoids analysis was *Y* = 0.5985*X* + 0.0575 with *R*^2^ = 0.9997 (RSD = 1.0%, *n* = 5), where *Y* is represented as absorbance, *X* is represented as concentration of rutin standard at 0.02–0.1 μg/mL. The extraction yield of total flavonoids was calculated using Equation (1):

(1) $$\mathrm{Extraction}\;\mathrm{yield}\;(\frac{\mathrm{mg}}{g})=\frac{\mathrm{Mass}\;\mathrm{of}\;\mathrm{total}\;\mathrm{flavonoids}\;\mathrm{determined}\;(\mathrm{mg})}{\mathrm{Mass}\;\mathrm{of}\;\mathrm{Apocynum}\;\mathrm{venetum}\;L.\mathrm{leaf}\;\mathrm{powder}\;(g)}$$

### 3.3. Aqueous Biphasic System

To a tube, a certain mass of salt and IL extract were added. The tube was centrifuged at 5000× *g* for 5 min after complete dissolution of salt. Two clear phases (one top IL-rich phase and the other bottom salt-rich phase) were observed with the volume of each phase being noted. Samples in top phase were withdrawn to another tube. The total flavonoids content in top phase was determined, and the mass in bottom phase was calculated by mass balance. The phase ratio (*R*) was defined according to Equation (2):

(2) $$R=\frac{V_{t}}{V_{b}}$$

where *V_t_* and *V_b_* were the volumes of the top and bottom phase, respectively. The partition coefficient (*K*) was calculated according to Equation (3):

(3) $$K=\frac{C_{t}}{C_{b}}$$

where *C_t_* was the total flavonoids content in top phase, *C_b_* was the total flavonoids content in bottom phase. The extraction efficiency (*E*) of total flavonoids in top phase was obtained according to Equation (4):

(4) $$E=\frac{K}{\left(K+\frac{1}{R}\right)}\times 100\%$$

The preconcentration factor (*F*) of the total flavonoids defined as given in Equation (5):

(5) $$F=\frac{C_{t}}{C_{initial}}$$

where *C_initial_* and *C_t_* represent the total flavonoids content in the initial aqueous solution before and after ABS extraction in the IL-rich phase, respectively.

### 3.4 Back-Extraction of Flavonoids and Recycle of IL

The flavonoids in the IL-rich phase were separated by back extraction using water immiscible organic solvents (dichloromethane, chloroform, *n*-butanol, isoamyl alcohol, diethyl ether, petroleum ether, ethyl acetate, and *n*-hexane) as reported by Bogdanov [42]. The flavonoids are back-extracted into the organic phase, while the ILs remain in aqueous phase and can thus be recycled.

## 4. Conclusions

In this work, IL-UAE coupled with ABS were applied for the extraction of flavonoids from *A. venetum* leaf using the IL [C_4_mim][N(CN)_2_] aqueous solution. The extraction conditions of IL concentration, liquid/solid ratio, and ultrasonic time were optimized by RSM, with further purification of the flavonoids by ABS. Using this developed methodology, we could obtain a preconcentration factor of 3.78%, with a remarkable extraction efficiency of 93.35%. After ABS, the back-extraction efficiency of flavonoids reached to 83.5% using *n*-butanol. The obtained results indicated that this IL-ABS coupled with ultrasound had the potentiality in the extraction and isolation of pharmaceutical components from various plant sources.

## Figures and Tables

**Table 1:** Experimental conditions for extraction yield using a three-factors/three-level Box-Behnken design.

Run	Factor A: Concentration of IL (g/mL)	Factor B: Liquid/Solid Ratio	Factor C: Ultrasonic Time (min)	Response Average Extraction Yield (mg/g)
1	0.20	15:1	20	7.84
2	0.40	15:1	20	6.59
3	0.20	25:1	20	8.50
4	0.40	25:1	20	6.61
5	0.20	20:1	15	8.10
6	0.40	20:1	15	6.24
7	0.20	20:1	25	7.21
8	0.40	20:1	25	6.39
9	0.30	15:1	15	7.86
10	0.30	25:1	15	8.23
11	0.30	15:1	25	7.15
12	0.30	25:1	25	7.79
13	0.30	20:1	20	8.77
14	0.30	20:1	20	8.79
15	0.30	20:1	20	8.89
16	0.30	20:1	20	8.76
17	0.30	20:1	20	8.85

**Table 2:** Analysis of variance for the response surface quadratic regression model.

Source	Degrees of Freedom	Sum of Squares	Mean Square	*F*-Value	*p*-Value Prob > *F*
Model	9	13.90	1.54	147.00	<0.0001
A	1	4.23	4.23	402.94	<0.0001
B	1	0.36	0.36	53.98	0.0006
C	1	0.45	0.45	42.49	0.0003
AB	1	0.1	0.1	9.75	0.0168
AC	1	0.27	0.27	25.73	0.0014
BC	1	0.018	0.018	1.73	0.2293
A^2^	1	5.09	5.09	484.63	<0.0001
B^2^	1	0.45	0.45	42.91	0.0003
C^2^	1	2.23	2.23	211.93	<0.0001
Residual	7	0.074	0.011		
Lack of fit	3	0.061	0.02	6.53	0.0508
Pure Error	4	0.012	3.12 × 10^−3^		
Cor total	16	13.98			

**Table 3:** Comparison of IL-UAE to conventional extraction methods.

Methods	Temperature (°C)	Time (h)	Yield (mg/g)	RSD (%)
IL-HRE(40%(*w*/*w*) IL)	100	6	7.31	3.5
Ethanol-SE(100%(*w*/*w*) ethanol)	80	6	6.45	2.8
Ethanol-UAE(40%(*w*/*w*) ethanol)	30	0.5	7.65	2.4
Ethanol-UAE(70%(*w*/*w*) ethanol)	30	0.5	7.88	1.8
Ethanol-UAE(100%(*w*/*w*) ethanol)	30	0.5	7.14	2.6
IL-UAE(40%(*w*/*w*) IL)	30	0.5	8.67	1.7
